# Molecular and Phylogenetic Characterization of Non-O157 Shiga Toxin-Producing *Escherichia coli* Strains in China

**DOI:** 10.3389/fcimb.2016.00143

**Published:** 2016-11-02

**Authors:** Xiangning Bai, Bin Hu, Yanmei Xu, Hui Sun, Ailan Zhao, Pengbin Ba, Shanshan Fu, Ruyue Fan, Yujuan Jin, Hong Wang, Qiusheng Guo, Xuebin Xu, Shan Lu, Yanwen Xiong

**Affiliations:** ^1^State Key Laboratory for Infectious Disease Prevention and Control, Collaborative Innovation Center for Diagnosis and Treatment of Infectious Diseases, National Institute for Communicable Disease Control and Prevention, Chinese Center for Disease Control and PreventionBeijing, China; ^2^Shandong Center for Disease Control and PreventionJinan, China; ^3^Longgang Center for Disease Control and PreventionShenzhen, China; ^4^Zigong Center for Disease Control and PreventionZigong, China; ^5^Suixian Center for Disease Control and PreventionSuixian, China; ^6^Shanghai Municipal Center for Disease Control and PreventionShanghai, China

**Keywords:** Shiga toxin, Shiga toxin-producing *E. coli*, non-O157 STEC, MLST, China

## Abstract

Shiga toxin-producing *Escherichia coli* (STEC) causes diarrhea and hemorrhagic colitis with life-threatening complications, such as hemolytic uremic syndrome. The aim of this study was to assess the molecular epidemiologic features of non-O157 STEC strains from different resources in China and illustrate the role of animal reservoirs or animal-derived foodstuffs in human STEC infections. A collection of 301 non-O157 STEC isolates from domestic and wild animals (i.e., cattle, goat, pig, yak, pika, and antelope), raw meats (i.e., beef, pork, mutton, chicken, and duck), diarrheal patients, and healthy carriers in different regions of China were selected in this study. Of the 301 analyzed STEC isolates, 67 serogroups, and 118 serotypes were identified; this included some predominant serogroups associated with human disease, such as O26, O45, O103, O111, and O121. Eighteen different combinations of *stx* subtypes were found. Eleven isolates carried the intimin gene *eae*, 93 isolates contained *ehxA*, and 73 isolates carried *astA*. The prevalence of other putative adhesion genes *saa, paa, efa1*, and *toxB* was 28.90% (87), 6.98% (21), 2.31% (7), and 1% (3), respectively. The phylogenetic distribution of isolates was analyzed by multilocus sequence typing (MLST). Ninety-four sequence types were assigned across the 301 isolates. A subset of isolates recovered from yak and pika residing in the similar wild environments, Qinghai-Tibetan plateau, showed similar genetic profiles and more tendencies to cluster together. Isolates from goat and mutton exhibited close genetic relatedness with those from human-derived isolates, providing evidence that transmission may have occurred locally within intraspecies or interspecies, and importantly, from animal reservoirs, or raw meats to humans. Comparing isolates in this study with highly virulent strains by MLST, along with serotyping and virulence profiles, it is conceivable that some of isolates from goat, yak, or raw meats may have potential to cause human diseases.

## Introduction

Shiga toxin-producing *Escherichia coli* (STEC) refers to an *E. coli* pathotype capable of producing either Shiga toxin 1 (Stx1), Shiga toxin 2 (Stx2), or both toxins. STEC has emerged as an important enteric foodborne zoonotic pathogen causing human gastrointestinal disease and has been implicated in sporadic cases and outbreaks of diarrhea, hemorrhagic colitis (HC), and hemolytic uremic syndrome (HUS) worldwide (Smith et al., 2014). Although STEC O157:H7 has been regarded as the predominant serotype and the main cause of STEC infection worldwide since the early 1980s, recent studies have shown that non-O157 STEC are emerging as important pathogens associated with numerous human infections as well as outbreaks of food-borne illnesses (Johnson et al., 2006; Käppeli et al., 2011). It was estimated that O157 was responsible for 35.9% of STEC infections, whereas non-O157 STEC was responsible for 64.1% of STEC infections in the United States (Scallan et al., 2011). Moreover, non-O157 STEC infections are responsible for majority of total STEC infections in Canada, Australia, Latin America, and Europe (Tozzi et al., 2003; Blanco et al., 2004; Brooks et al., 2005). To date, more than 200 non-O157 STEC serotypes have been identified and associated with human illness worldwide (Johnson et al., 2006; Coombes et al., 2008). Nevertheless, most studies have been focused on the serotype O157:H7 and the top six non-O157 serogroups (i.e., O26, O45, O103, O111, O121, and O145) (Conrad et al., 2014); this is likely because there is no standard detection method covering all non-O157 STEC serotypes due to the high genotypic and phenotypic diversity. Therefore, the public health significance of non-O157 STEC is likely to be underestimated.

Shiga toxin is derived from *Shigella dysenteriae*, which was first described by Kiyoshi Shiga in 1898. The production of Stx is considered to be the most important virulence factor associated with STEC. In humans, Stx binds to the glycosphingolipid Gb3, a molecule that is mostly observed in kidney epithelium and endothelium as well as microvascular endothelial cells in intestinal lamina propria, and damages intestinal epithelial cells and kidneys resulting in HC and HUS (Melton-Celsa, 2014). Stxs are classified into two major types, Stx1 and Stx2, which are encoded by the *stx*_1_ and *stx*_2_ genes, respectively. Stx1 and Stx2 are further categorized into several subtypes, according to the classification proposed by Scheutz et al. The *stx*_1_ gene consists of three subtypes, *stx*_1a_, *stx*_1c_, and *stx*_1d_; while seven *stx*_2_ subtypes (i.e., *stx*_2a_, *stx*_2b_, *stx*_2c_, *stx*_2d_, *stx*_2e_, *stx*_2f_, and *stx*_2g_) have been identified (Scheutz et al., 2012). Specific *stx* subtypes are associated with human infections; for example, *stx*_2a_, *stx*_2c_, and *stx*_2d_ are often isolated from patients with HUS (Fuller et al., 2011; Melton-Celsa, 2014; Fruth et al., 2015). Whereas, others are related to nonhuman animal infections; for example, *stx*_2e_ is associated with edema disease in pigs (Meisen et al., 2013; Tseng et al., 2014).

In addition to Stx, there are other virulence factors that contribute to the pathogenesis of STEC (Farfan and Torres, 2012). The intimin, encoded by the *eae* gene on the locus of enterocyte effacement (LEE), can lead to the formation of attaching and effacing (A/E) lesions (Elliott et al., 2000). However, the emergence of human infections linked to LEE negative STEC strains indicates that this pathogenicity island is not the only factor responsible for adherence (Galli et al., 2010). Several other proteins have been proposed to be putative adhesion factors. ToxB is a protein that is identified from a 93-kb plasmid pO157 and is required for full adherence of the O157:H7 strain Sakai (Tatsuno et al., 2001). Saa is an autoagglutinating adhesin unique to LEE-negative STEC strains (Paton et al., 2001). Efa1 is associated with STEC serotypes that are linked to epidemic and/or serious diseases (Nicholls et al., 2000; Karmali et al., 2003). Paa is involved in the intimate attachment of bacteria to enterocytes and induces typical A/E lesions in pigs (Vidotto et al., 2013). Other virulence determinants have also been identified in STEC strains; for instance, the enterohemolysin, which is encoded by the *ehxA* gene on a 60-MDa virulence plasmid (Jiang et al., 2015), and the enteroaggregative *E. coli* (EAEC) heat-stable enterotoxin, which is encoded by the *astA* gene (Nishikawa et al., 2002).

Cattle are reported to likely be the most important reservoir of non-O157 STEC strains (Bibbal et al., 2015); however, other animals, such as sheep, goats, swine, birds, wild animals, and humans, can also harbor STEC strains in their digestive tracts (Mora et al., 2012; Chandran and Mazumder, 2013; Singh et al., 2015). In our previous study, we investigated STEC in some domestic and wild animals, as well as foodstuffs of animal origin, in China (Bai et al., 2013, 2015, 2016; Meng et al., 2014). STEC strains have also been isolated from diarrheal patients in China (Xiong et al., 2012; Chen et al., 2014; Wang et al., 2015; Yu et al., 2015). However, little knowledge is available on the molecular and phylogenetic properties of non-O157 STEC from various sources in China, and the role of animal reservoirs or animal-derived foodstuffs in causing human non-O157 STEC infections remains unknown. The aim of the study was a further characterization of non-O157 STEC isolates recovered from multiple sources including domestic and wild animals (i.e., cattle, goat, pig, yak, pika, and antelope), raw meats (i.e., beef, pork, mutton, chicken, and duck), diarrheal patients, and healthy carriers in different regions of China.

## Materials and methods

### Bacterial strains

In total, 301 non-O157 STEC isolates were collected from various sources in China; of these, 54, 22, 93, and 63 were isolated from the yak, pika, pig, and raw meats sources reported in our previous studies (Bai et al., 2013, 2015, 2016; Meng et al., 2014). Additionally, 2, 12, 28, 24, and 3 strains were isolated from antelope, cattle, goat, diarrheal patients, and healthy carriers respectively (Table 1); these strains were isolated using previously described methods (Bai et al., 2016). Briefly, all samples were enriched in *E. coli* broth (Land Bridge, Beijing, China) and screened for *stx* genes by a duplex PCR assay; the primers used for detecting *stx*_1_ and *stx*_2_ genes are listed in Table 2. Enriched samples that tested positive for *stx*_1_ and/or *stx*_2_ genes were plated on CHROMagar™ ECC agar (CHROMagar, Paris, France), CHROMagar™ STEC agar (CHROMagar, Paris, France), and MacConkey agar (Oxoid, Hampshire, UK) and incubated overnight at 37°C. The following colonies were picked and screened for the presence of *stx*_1_ and/or *stx*_2_ genes by single colony duplex PCR assay: blue or colorless round moist *E. coli*-like colonies on ECC agar; mauve colonies on STEC agar; and pink or red colonies on MacConkey agar. Each *stx*-positive isolate was confirmed to be *E. coli* by biochemical tests using the commercially available API 20E system (bioMérieux, France; http://www.biomerieux.com). The prevalence of non-O157 STEC in different sources is shown in Table 1.

**Table 1:** **Non-O157 STEC isolates used in this study and origin of isolation**.

**Source**	**Location**	**Sampling year**	**No. of samples**	**No. of isolates**	**No. MLST analyzed**
Yak	Yushu Tibetan	2012	728	128	54
Pika	Yushu Tibetan	2012, 2013, 2015	1116	22	22
Antelope	Hoh Xil	2014	–^a^	2	2
Cattle	Heilongjiang and Shandong	2009, 2012, 2015	440	12	12
Goat	Henan	2011	–^a^	28	28
Pig	Chongqing, Beijing, and Guizhou	2011–2012	1003	93	93
Raw meat	Beijing and Sichuan	2013–2014	853	63	63
Diarrheal patient	Henan, Shenzhen, Shanghai, and Sichuan	2010–2014	870	24^b^	24
Healthy carrier	Qinghai and Shenzhen	2013–2014	1058	3	3
Total			6068	375	301

a *The number of samples was not applicable*.

b *Among the 24 isolates, 11 were recovered from the 870 diarrheal patients from Henan province, and 13 were obtained from local centers for disease control and prevention of Shenzhen city, Shanghai city, and Sichuan province. The number of samples was not applicable*.

**Table 2:** **PCR primers used for the detection of STEC virulence or adherence genes (Bai et al., 2016)**.

**Target**	**Primer forward/Primer reverse (5′-3′)**	**Amplicon size (bp)**	**Annealing temperature (°C)**
*stx*_1_	AAATCGCCATTCGTTGACTACTTCT/	370^a^	58
	TGCCATTCTGGCAACTCGCGATGCA		
*stx*_2_	CAGTCGTCACTCACTGGTTTCATCA/	283^a^	58
	GGATATTCTCCCCACTCTGACACC		
*stx*_2_	ATGAAGTGTATATTATTTAAATGG/	1260^b^	55
	TCAGTCATTATTAAACTGCAC		
*eae*	TCAATGCAGTTCCGTTATCAGTT/	482	58
	GTAAAGTCCGTTACCCCAACCTG		
*efa1*	GAGACTGCCAGAGAAAG/	479	51
	GGTATTGTTGCATGTTCAG		
*saa*	CGTGATGAACAGGCTATTGC/	119	52
	ATGGACATGCCTGTGGCAAC		
*paa*	ATGAGGAAACATAATGGCAGG/	350	60
	TCTGGTCAGGTCGTCAATAC		
*astA*	CCATCAACACAGTATATCCGA/	111	55
	GGTCGCGAGTGACGGCTTTGT		
*ehxA*	GGTGCAGCAGAAAAAGTTGTAG/	1551	57
	TCTCGCCTGATAGTGTTTGGTA		
*toxB*	ATACCTACCTGCTCTGGATTGA/	602	55
	TTCTTACCTGATCTGATGCAGC		

a *Primers for detection of stx_1_ and stx_2_ using duplex PCR*.

b Primers used for amplifying and sequencing the full length of stx_2._

### Serotyping

The O antigen was initially screened using the O-genotyping PCR method to identify and classify the *E. coli* O serogroups (Iguchi et al., 2015). The complete *E. coli* O antisera (O1-O188; Statens Serum Institut, Hillerød, Denmark) were used to confirm the PCR results. The isolates were referred as O-untypable if they did not react with any O antisera. The entire coding sequence of *fliC* was amplified by PCR using the primers: F-FLIC1 (5′-ATGGCACAAGTCATTAATACCCAAC-3′) and R-FLIC2 (5′-CTAACCCTGCAGCAGAGACA-3′) (Fields et al., 1997). Then, the PCR products were sequenced and compared to a publicly available CGE SerotypeFinder database (http://cge.cbs.dtu.dk/services/) to determine the H type of each isolate (Joensen et al., 2015). The isolate was H-untypable if *fliC* was negative by PCR.

### *stx* subtyping and detection of virulence factors

The *stx*_1_ and/or *stx*_2_ subtypes of all non-O157 STEC isolates were determined by PCR-based subtyping method (Scheutz et al., 2012). The full length of *stx*_1_ and/or *stx*_2_ gene of some STEC isolates was amplified using previously reported primers (Bai et al., 2016), and the gene was sequenced to verify the PCR-based subtyping results. The neighbor-joining cluster analysis was employed to assign subtypes or variants (Scheutz et al., 2012).

Except for the *stx* genes, all 301 non-O157 STEC isolates were subjected to PCR for detection of the intimin-encoding gene *eae*, the putative adhesin genes *efa1, saa, paa*, and *toxB*, and the virulence genes *ehxA* and *astA* (Table 2).

### Multilocus sequence typing

Multilocus sequence typing (MLST) was used to characterize phylogenetic relationships and assess the potential risks for human infection. Defined fragments of the seven housekeeping genes (i.e., *adk, icd, fumC, recA, mdh, gyrB*, and *purA*) were amplified and sequenced according to the *E. coli* MLST website (http://mlst.warwick.ac.uk/mlst/dbs/Ecoli). Sequences types (STs) for each isolate were assigned based on the allelic profile of the seven housekeeping genes. A neighbor-joining tree was constructed by MEGA 6 based on the concatenated sequences of the seven housekeeping genes, and was used to analyze the phylogenetic relationships among strains. STs of isolates from this study were then compared with those from highly pathogenic STEC strains, including the STs of the HUS-associated enterohemorrhagic *E. coli* (HUSEC) collection (www.ehec.org) (Mellmann et al., 2008), and human STEC STs of O157 and the top six non-O157 serogroups (i.e., O26, O45, O103, O111, O121, and O145 retrieved from the *E. coli* MLST website). A minimum spanning tree (MST) based on these STs was generated using the BioNumerics software.

## Results

### Serogroups and serotypes

There were 118 distinct serotypes from the 301 isolates in this study; this included 67 O serogroups and 25 H types. Sixty-three isolates were O-untypable (ONT) and five isolates were H-untypable (HNT). The predominant were serotypes ONT:H30 and O20:H30, which accounted for 30 and 21 isolates, respectively; other prevalent serotypes were: O2:H32 (10 isolates), ONT:H21 (9 isolates), O21:H25 (9 isolates), O128:H2 (8 isolates), O103:H8 (8 isolates), O2:H45 (7 isolates), O130:H8 (6 isolates), O117:H21 (6 isolates), O8:H16 (5 isolates), and O176:H4 (5 isolates). Nine serotypes (i.e., ONT:H20, ONT:H19, O91:H4, O91:H14, O8:H9, O8:H19, O172:H30, O104:H7, and O100:H20) contained four isolates each; 11 serotypes (i.e., ONT:H8, ONT:H7, O98:H30, O81:H31, O8:H2, O78:H8, O74:H8, O26:H11, O22:H8, O117:H8, and O100:H19) contained three isolates each; 22 serotypes contained two isolates each; while 64 serotypes contained only one isolate each (Table S1).

The majority of non-O157 STEC strains that are a threat to human health are associated with six specific serogroups (i.e., O26, O45, O103, O111, O121, and O145). In this study, four isolates from diarrheal patients were assigned to these serogroups/serotypes (three were O26:H11 and one was O111:H8). The serotypes O103:H8 and O45:H2 were detected in eight and two goat isolates, respectively; two beef isolates were assigned to serotype O103:H25, and serotype O121:H10 was found in one pork isolate. None of the STEC isolates from antelopes, pikas, yaks, pigs, cattle, or healthy carriers belonged to the six top serogroups (Table S1).

### Shiga toxin and presence of other virulence genes

Of the 301 STEC strains characterized, 79 (26.25%) were *stx*_1_ positive only, 192 (63.79%) were *stx*_2_ positive only, and 30 (9.97%) were both *stx*_1_ and *stx*_2_ positive. Overall, three *stx*_1_ subtypes (i.e., *stx*_1a_, *stx*_1c_, and *stx*_1d_) and six *stx*_2_ subtypes (i.e., *stx*_2a_, *stx*_2b_, *stx*_2c_, *stx*_2d_, *stx*_2e_, and *stx*_2g_) were detected; this resulted in a total of 18 different *stx*_1_ and *stx*_2_ subtype combinations. Among these subtype combinations, *stx*_2e_ was most prevalent (121 isolates), followed by *stx*_1a_ (43), *stx*_1c_ (35), *stx*_2b_ (26), *stx*_2d_ (20), *stx*_1a_, and *stx*_2d_ (10), and *stx*_2g_(10); this accounted for a total of 265 strains (88.04%) (Table 3).

**Table 3:** *****stx*** prevalence in relation to origin of isolation**.

***stx* types and subtypes (no. of strains, %)**	**No. of strains isolated from:**									
	**Yak (*n* = 54)**	**Pika (*n* = 22)**	**Antelope (*n* = 2)**	**Cattle (*n* = 12)**	**Goat (*n* = 28)**	**Pig (*n* = 93)**	**Raw meat (*n* = 63)**	**Diarrheal patient (*n* = 24)**	**Healthy carrier (*n* = 3)**	**Total (*n* = 301)**
*stx*_1_ (79, 26.25%)	11	5	2	0	19	0	20	21	1	79
*stx*_2_ (192, 63.79%)	35	14	0	10	4	93	32	3	1	192
*stx*_1_+*stx*_2_ (30, 9.97%)	8	3	0	2	5	0	11	0	1	30
*stx*_2e_ (121, 41.20%)	0	0	0	0	1	93	25	2	0	121
*stx*_1a_ (43, 14.29%)	11	5	2	0	9	0	6	10	0	43
*stx*_1c_ (35, 11.63%)	0	0	0	0	10	0	13	11	1	35
*stx*_2b_ (26, 8.64%)	20	5	0	0	0	0	1	0	0	26
*stx*_2d_ (20, 6.64%)	5	7	0	4	2	0	1	0	1	20
*stx*_1a_+*stx*_2d_ (10, 3.32%)	5	3	0	1	1	0	0	0	0	10
*stx*_2g_ (10, 3.32%)	3	0	0	5	1	0	0	1	0	10
*stx*_1c_+*stx*_2b_ (9, 2.99%)	0	0	0	0	0	0	9	0	0	9
*stx*_2a_ (8, 2.66%)	3	1	0	0	0	0	4	0	0	8
*stx*_1c_+*stx*_2d_ (4, 1.33%)	0	0	0	0	4	0	0	0	0	4
*stx*_1a_+*stx*_2a_ (3, 0.99%)	1	0	0	1	0	0	1	0	0	3
*stx*_1a_+*stx*_2b_ (3, 0.99%)	1	0	0	0	0	0	1	0	1	3
*stx*_2c_ (3, 0.99%)	1	0	0	1	0	0	1	0	0	3
*stx*_2a_+*stx*_2c_ (2, 0.66%)	2	0	0	0	0	0	0	0	0	2
*stx*_1d_ (1, 0.33%)	0	0	0	0	0	0	1	0	0	1
*stx*_2a_+*stx*_2d_ (1, 0.33%)	0	1	0	0	0	0	0	0	0	1
*stx*_2a_+*stx*_2b_ (1, 0.33%)	1	0	0	0	0	0	0	0	0	1
*stx*_1a_+*stx*_2a_+*stx*_2b_ (1, 0.33%)	1	0	0	0	0	0	0	0	0	1

Isolates from yaks, pikas, goats, raw meats, and healthy carriers had different *stx* subtypes and combinations. All of the 107 pig-derived isolates (93 from fecal samples and 14 from pork) possessed only *stx*_2e_. Two isolates from antelope were only *stx*_1a_. The majority of cattle isolates (10/12, 83.33%) harbored only *stx*_2_; of these, *stx*_2d_ and *stx*_2g_ were the dominant subtypes. The isolates from diarrheal patients were largely dominated by only *stx*_1_ (21/24, 87.5%); of these, 10 were the *stx*_1a_ subtype and 11 were the *stx*_1c_ subtype (Table 3).

For the other virulence genes: 11 isolates from diarrheal patients (5), beef (3), cattle (1), yak (1), and mutton (1) were *eae* positive; all of these also had high virulent *stx* profiles (eight contained only *stx*_1a_, two carried only *stx*_2c_, and one harbored only *stx*_2a_). *ehxA* was detected in 93 isolates covering all reservoirs investigated in this study, and the 11 *eae* positive isolates were all positive for *ehxA*. Seventy-three isolates contained *astA*; the majority (51/73, 69.86%) of these were pig-derived. The prevalence of the adhesion-associated genes *saa, paa*, and *efa1* was 28.90% (87), 6.98% (21), and 2.31% (7), respectively (Table 4). Notably, three isolates from diarrheal patients (STEC406, STEC411, and STEC416) assigned to the highly virulent serotype O26:H11 were *toxB* positive, and all of them possessed *eae, ehxA, efa1*, and *paa* (Table S1).

**Table 4:** **Virulence genes in non-O157 STEC isolates**.

**No. of isolates**	***eae***	***ehxA***	***astA***	***efa1***	***saa***	***paa***	***toxB***
1	+	+	−	+	+	+	−
1	+	+	−	+	+	−	−
3	+	+	−	+	−	+	+
1	+	+	−	+	−	+	−
3	+	+	−	−	−	+	−
2	+	+	−	−	−	−	−
1	−	+	+	−	−	+	−
1	−	+	+	−	−	−	−
1	−	+	−	+	−	+	−
64	−	+	−	−	+	−	−
2	−	+	−	−	−	+	−
13	−	+	−	−	−	−	−
2	−	−	+	−	+	−	−
6	−	−	+	−	−	+	−
63	−	−	+	−	−	−	−
19	−	−	−	−	+	−	−
3	−	−	−	−	−	+	−

*^+^Positive*.

*^−^Negative*.

### Phylogenetic analysis of non-O157 STEC by MLST

**Figure 1:**
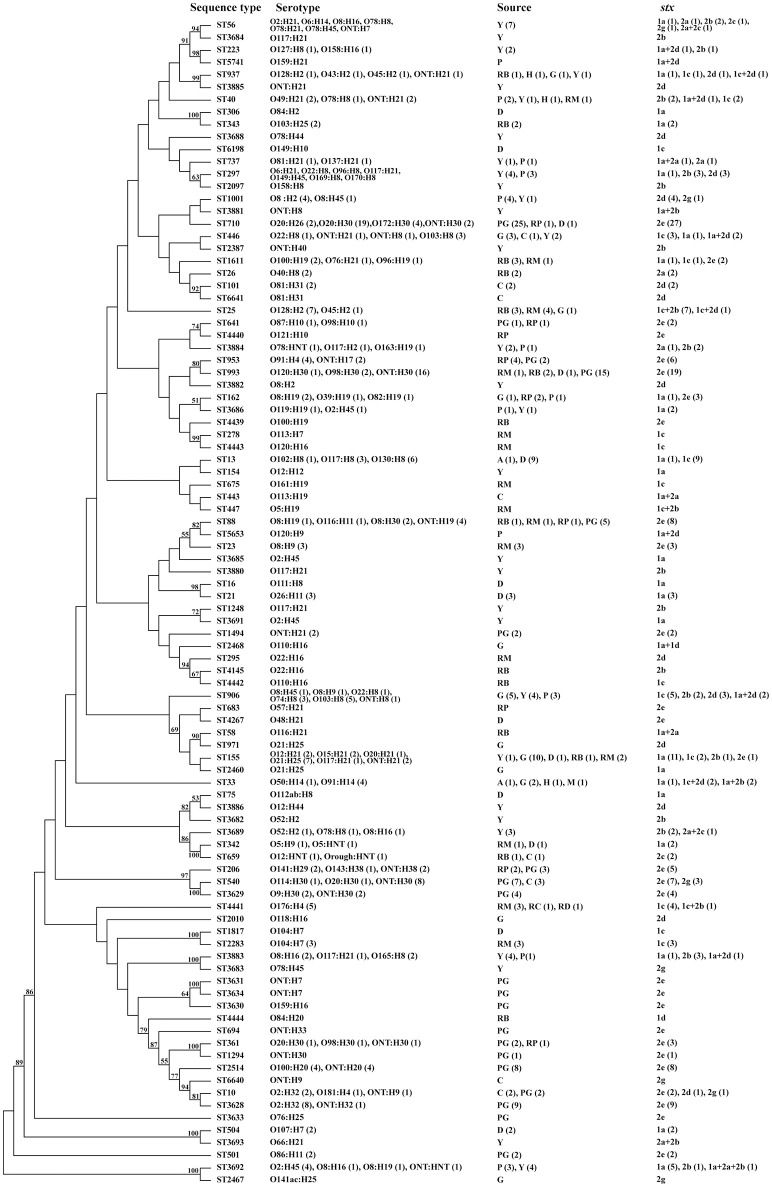
**Phylogenetic tree based on concatenated sequences of seven housekeeping genes of all 301 isolates by the Neighbor−joining method using MEGA 6**. Numbers on the tree indicate bootstrap values calculated for 1000 subsets for branch points >50%. For sequence types that consisted of more than one isolates, the number of isolates for individual profiles is provided in brackets. ^a^Origin of isolation: Y, yak; P, pika; A, antelope; C, cattle; G, goat; PG, pig; D, diarrheal patients; H, healthy carriers; RB, raw beef; RM, raw mutton; RP, raw pork; RC, raw chicken; and RD, raw duck.

Ninety-four sequence types (STs) were obtained from the 301 non-O157 STEC isolates. ST710 (27 isolates) and ST993 (19 isolates) were most common, followed by ST155 (15 isolates), ST906 (12 isolates), ST13 (10 isolates), ST540 (10 isolates), and ST3628 (9 isolates). Three STs (ST25, ST88, and ST2514) contained eight isolates each; ST56, ST297, and ST3692 contained seven isolates each; ST446 and ST953 contained six isolates each. Further, six STs (ST33, ST40, ST206, ST1001, ST3883, and ST4441) were detected in five isolates each; five STs (ST10, ST162, ST937, ST1611, and ST3629) contained four isolates each; six STs contained three isolates each; while 12 STs contained two isolates each and 50 STs were found only once (Figure 1).

Isolates from various sources or same host were widely distributed across a phylogenetic tree; this demonstrates the high genetic diversity. There were isolates recovered from yak and pika samples in the same unique ecosystem (Qinghai-Tibetan plateau) that shared same STs (i.e., ST40, ST297, ST773, ST906, ST1001, ST3686, ST3692, ST3883, and ST3885). Three subsets of STs (i.e., ST56, ST3684, ST223, and ST5741; ST737, ST297, and ST2097; ST1001 and ST3881) from both yak and pika were grouped on similar branches. Notably, four isolates of ST3692, three from yaks and one from pika, shared the same serotype (O2:H45), *stx* subtype (*stx*_1a_ only), and virulence gene composition (Figure 1 and Table S1). ST88, ST206, ST361, ST641, ST710, and ST953 contained isolates from both pig feces and pork, all of which only carried *stx*_2e_. A subset of STs (ST1611, ST26, ST101, and ST6358) within a cluster contained isolates from cattle feces and beef. The 24 isolates from diarrheal patients assigned to 13 STs were scattered throughout the phylogenetic tree. Isolate STEC408 from a diarrheal patient sampled in Shanghai city shared the same sequence type (ST155), serotype (O21:H25), *stx* subtype (*stx*_1a_ only) and virulence profiles with four isolates from goats sampled in Henan province (STEC012, STEC013, STEC014, and STEC021). Similarly, isolate STEC409 from a diarrheal patient in Shanghai city had the same sequence type (ST993), serotype (ONT:H30), and *stx* subtype (*stx*_2e_ only) as 13 pig-derived isolates sampled in Beijing city and Chongqing city, all of which possessed none of the virulence genes tested (Figure 1 and Table S1). A subset of STs (ST33, ST40, and ST937) isolated from healthy carriers were also found in isolates from raw meats, goat, yak, pika and antelope. Of these, ST33 of a healthy carrier (STEC435) in Qinghai province had the same serotype O91:H14 as isolated from goat and mutton; it also had the same *stx* subtype as the mutton isolate STEC362. ST40 from a healthy carrier (STEC438) in Shenzhen city had the same serotype and *stx* subtype as a mutton isolate (STEC317) sampled in Beijing city (Figure 1 and Table S1).

### Comparison of STEC isolates with highly pathogenic STEC isolates

**Figure 2:**
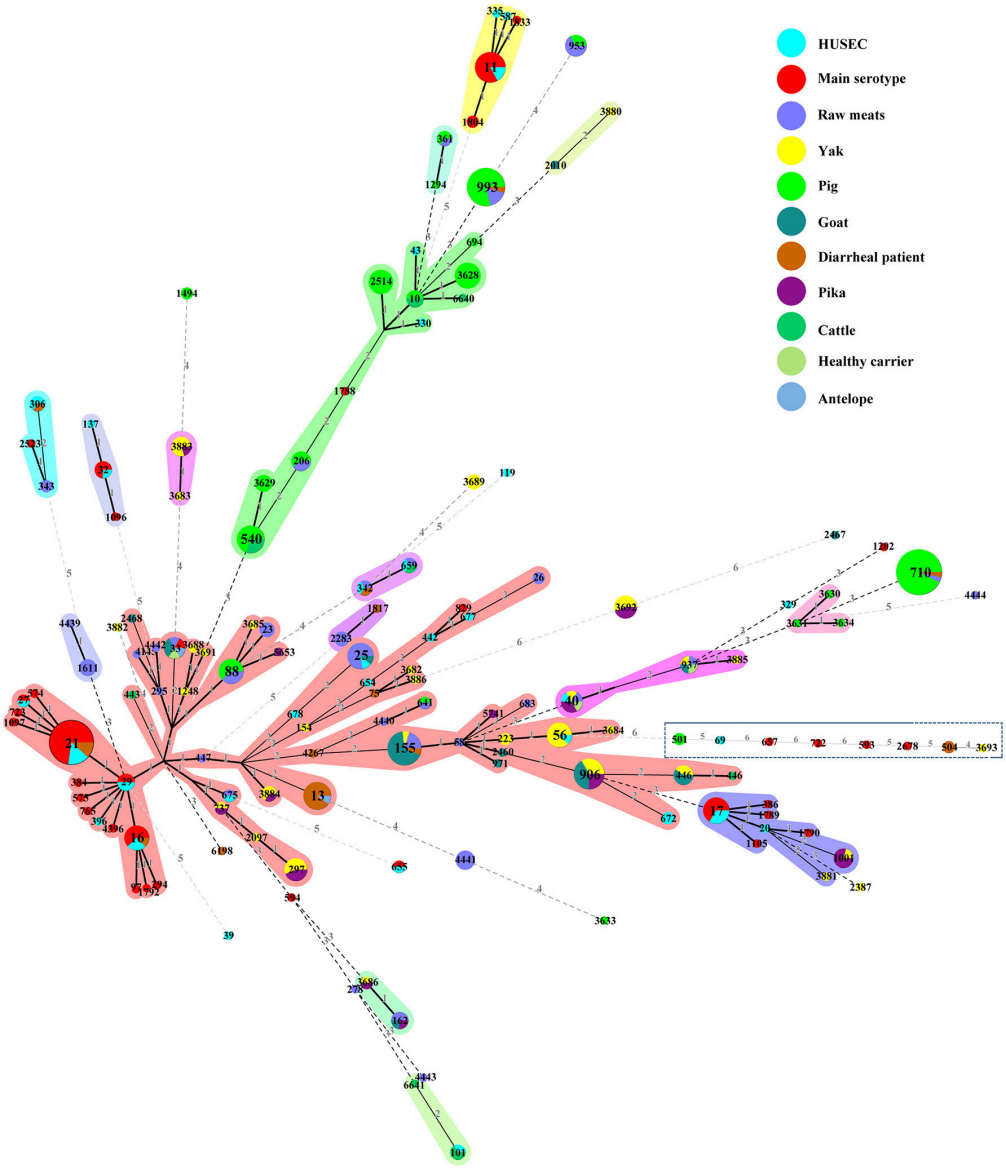
**Minimum spanning tree of 94 STs from this study, 32 STs from the HUSEC collection (blue), and 36 STs from human STEC STs of serogroups O26, O45, O103, O111, O121, O145, and O157 in the ***E. coli*** MLST database (red)**. Each circle represents an ST with the size being proportional to the number of isolates. The colors for the slices of the pie represent the sources of the isolates (see labeling in the upper right corner). The numbers on the connecting lines represent the number of allelic differences between two STs.

We constructed an MST containing 94 STs of various non-O157 STEC in our study, 32 STs from the HUSEC collection, and 36 human STEC STs of O157 and the top six serogroups from the *E. coli* MLST database to assess the potential risk for human infection (Figure 2). Four STs (i.e., ST16, ST21, ST306, and ST342) from diarrheal patients were observed in human STECs; ST21 contained three O26:H11 isolates that carried the *stx*_1a_, *eae, ehxA, toxB, efa1*, and *paa* genes. Isolates of ST40 from healthy carrier, raw mutton, pika, and yak samples were found in the HUSEC collection. Among these, the isolate from yak (MN1205-30) carried *stx*_1a_, *stx*_2d_, *ehxA*, and *saa*; the isolate from mutton (STEC317) harbored *stx*_1c_ and *saa*; while none of the three isolates (two from pika and one from healthy carrier) possessed any of the virulence genes tested in this study except for *stx* (Figure 2 and Table S1). Moreover, ST25 from the goat and raw meat (beef and mutton) isolates, ST56 from the yak isolates, ST342 and ST675 from the raw mutton isolates shared the same STs with HUSEC collection (Figure 2). The isolates from these reservoirs had variability in the presence of virulence genes; particularly, ST25 from a goat isolate (STEC006) was assigned to the pathogenic serotype O45:H2, carrying *stx*_1c_, *stx*_2d_, and *ehxA* genes. Five isolates from antelope (STEC477), goat (STEC007 and STEC009), healthy carrier (STEC435), and raw mutton (STEC362) had the same ST (ST33) as human STECs; four of these isolates were the serotype O91:H14, which is one of the most common serotypes in human pathogenic *eae*-negative STEC strains; while one isolate from antelope (STEC477) was assigned to serotype O50:H14 (Table S1).

## Discussion

In this study, we performed a detailed analysis of the molecular epidemiology of non-O157 STEC in China, using a collection of 301 STEC isolates recovered from fecal samples (i.e., yak, pika, antelope, pig, cattle, goat, diarrheal patients, and healthy carriers) and raw meats. The prevalence of STEC in yak, pika, pig, cattle, and raw meats samples was 17.58, 1.99, 9.27, 2.73, and 7.4%, respectively (Bai et al., 2013, 2015, 2016; Meng et al., 2014); the number of goat and antelope samples was not applicable (Table 1). Additionally, we recovered 11 isolates from 870 diarrheal patients. These samples had a positive rate of 1.26%, which was lower than the rate for animal samples, but slightly higher than the rate reported in other studies in China (0.1–0.57%) (Chen et al., 2014; Wang et al., 2015; Yu et al., 2015). This is the first investigation of STEC prevalence in healthy human population in China. Only 3 isolates were recovered from 1058 fecal samples (0.28%) of healthy carriers, indicating that STEC are uncommon in the healthy human population. Notably, it was also the first report worldwide on STEC recovery from Tibetan antelopes (*Pantholops hodgsonii*) living on plateaus with elevations between 3500 and 5500 m, a low partial pressure of oxygen, and high ultraviolet radiation levels, and follows up on our previous studies of STEC in yak and pika residing in the similar extremely harsh wild environments (Bai et al., 2013, 2015). This is of interest as it does indicate that wild animals in these environments are natural reservoirs of STEC, and extending our knowledge of the reservoir host range of STEC.

A high diversity of serotypes was observed among isolates. Some serotypes tended to be reservoirs-specific; for example, serotypes O20:H30, O2:H32, and O100:H20 were only detected in pig isolates; serotypes O15:H21, O45:H2, and O103:H8 were only detected in goat isolates; serotypes O78:H8, and O117:H21 were only detected in yak isolates. The O serogroups could not be determined for 20.93% (63/301) of the isolates, which may represent emerging serogroups or serotypes; however, the potential for causing human infection remains unknown. Even though hundreds of non-O157 serotypes have been described, serotypes O26, O45, O103, O111, O121, and O145 have been suggested to be the most important for human health (Brooks et al., 2005). In this study, there were isolates recovered from diarrheal patients, goat, and pork that were assigned to these highly pathogenic serogroups, including O26, O45, O103, O111, and O121. However, none of the STEC isolates from antelope, pika, yak, pig, or healthy carriers belonged to these serotypes; this implies that most animals and healthy carriers are not reservoirs of the predominant serotypes. Whereas, some of the serotypes identified in this study, including O8:H2 from pika, O8:H19 from pork and yak, O22:H8 from yak and cattle, O118:H16 from goat, O128:H2 from beef and mutton, and O163:H19 from pika have been previously isolated from human infections with hemolytic uremic syndrome (Hussein, 2007).

Shiga toxin subtypes have been implicated in severe outcome of STEC infection. Stx2 is more frequently associated with disease than Stx1, while Stx2a, Stx2c, and Stx2d are more commonly associated with HC and HUS. The remaining subtypes are only found in patients with uncomplicated diarrhea (Fuller et al., 2011; Melton-Celsa, 2014; Fruth et al., 2015). Swine STEC isolates commonly produce Stx2e, which may cause edema disease in weaned pigs (Meisen et al., 2013; Tseng et al., 2014). In this study, we found some highly pathogenic *stx* compositions (i.e., *stx*_1a_, *stx*_2a_, *stx*_2c_, *stx*_2d_, *stx*_1a_+*stx*_2a_, *stx*_2a_+*stx*_2c_, *stx*_2a_+*stx*_2d_, and *stx*_1a_+*stx*_2a_+*stx*_2b_) that were linked to severe human illness. Almost all animal isolates, with the exception of pig-derived isolates, support our hypothesis that pig-derived STEC isolates have a low potential for causing human disease (Meng et al., 2014). The combination of both *stx* and *eae* genes has been associated with enhanced virulence and increased severity of clinical infections in humans (Werber et al., 2003). We observed 11 isolates (five from diarrheal patients, four from raw meats, one from cattle, and one from yak) that carried *eae*; all were *ehxA* positive. Notably, *toxB* was detected in three O26:H11 isolates from diarrheal patients and *efa1* was positive in seven isolates; these isolates belonged to the serotypes O26:H11 (3), O111:H8, O5:HNT, O5:H9, and O78:HNT. This founding supports the notion that the putative adhesion genes *toxB* and *efa1* are present mainly in isolates of highly prevalent and pathogenic serotypes that are *eae* positive (Toma et al., 2004); thus, this is an indicator of high risk for human illness.

MLST has been proposed as an adequate tool for producing genetic profiles for a vast number of isolates, especially under non-epidemic circumstances (Ferdous et al., 2016). In this study, a substantial genetic diversity was observed in 301 non-O157 STEC isolates collected from various sources. Although there were no clear phylogenetic clusters for the majority of isolates and no clear correlations were either observed between ST, serotype or *stx* subtype, the strains did cluster based on geographic location or animal species. We observed that a subset of isolates recovered from yak and pika in the same unique ecosystem, the Qinghai-Tibetan plateau, shared same STs and were more likely to cluster together. Thus, it is possible that some isolates persist in specific environments and are transmitted locally within intraspecies and interspecies. A similar correlation was also observed in isolates derived from the same animal species regardless of whether they arise from feces or animal meat, e.g., feces of pig and pork or feces of cattle and beef, indicating that most STEC infections due to animal-derived foodstuffs might be traced back to the food producing animals as a specific source, instead of an entry from environmental or human sources along the food chain. Notably, isolates obtained from diarrheal patients did not belong to a specific phylogenetic cluster and were scattered throughout the phylogenetic tree. Furthermore, isolates from goats, pigs, and raw meats were genetically similar to those from human-derived isolates based on MLST, serotypes and virulence profiles, regardless of geographic locations. This also supports the notion of STEC transmission from livestock or raw meats to humans; nevertheless, the transmission route is still unclear and warrants further investigation.

An analysis of non-O157 STEC isolates in this study against the HUSEC collection, and human STECs of O157 and the top six serogroups revealed that some STEC isolates from diarrheal patients, healthy carriers, goats, pikas, yaks, and raw meats were closely related to pathogenic STEC isolates. Moreover, when combined with serotyping and virulence analysis, it is clear that isolates from diarrheal patients, goats, yaks, and raw meats are more likely to possess highly pathogenic serotypes that have been associated with severe diseases including O26:H11, O111:H8, O45:H2, O91:H14, and O8:H19 (Hussein, 2007; Bielaszewska et al., 2009), as well as combinations of highly pathogenic *stx* subtypes and virulence gene panels. These data suggest that diarrheal patients, goats, yaks, and raw meats are the important reservoirs of pathogenic STEC strains; while, isolates from antelopes, pikas, pigs, cattle, and healthy carriers likely pose low risks to human health. Given that non-O157 STEC strains are oral-fecal organisms, it is urgent to monitor the local human population for STEC infections, which may be acquired by direct contact with infected patients, wild, or domestic animals as well as consumption of contaminated animal-derived foodstuffs.

## Conclusions

To the best of our knowledge, this is the first and largest survey to systematically analyze the molecular and phylogenetic features of non-O157 STEC strains in China. Our survey indicates that non-O157 STEC are widely distributed across multiple sources and regions in China. MLST analysis, serotyping, *stx* subtyping, and virulence gene profiling suggest that STEC from different resources across China are highly genetically diverse, that transmission may occur within intraspecies or interspecies, and there is potential for human infections to originate from animal reservoirs or animal-derived foodstuffs. The pathogenicity varied across isolates from different sources. Further work should involve investigation of the epidemiological role of animal reservoirs and animal-derived foodstuffs in the maintenance of STEC, and the genetic markers that related to pathogenicity to determine the direction of transmission and differentiate low- and high-risk non-O157 STEC infections.

## Author contributions

XB, SL and YX designed the project; XB, YX, YJ, HW, QG, BH, SL, and XX carried out the sampling work; XB, BH, YXu, AZ, HS, PB, SF, and RF carried out the experiments and generated data; XB, SL, and YX analyzed data and drafted the manuscript.

### Conflict of interest statement

The authors declare that the research was conducted in the absence of any commercial or financial relationships that could be construed as a potential conflict of interest.

## Supplementary material

Table S1 **Characterization of 301 non−O157 STEC isolates analyzed in this study**.
